# Unveiling Bacterial Diversity in Portuguese Red Wine Effluents Through a Metagenomic Approach

**DOI:** 10.3390/microorganisms13092192

**Published:** 2025-09-19

**Authors:** Ana Gabriela Gomes, Ana Cláudia Sousa, João S. Carreira, Alberto Oliveira, Marta C. Justino, Carla Amarelo Santos

**Affiliations:** 1 MARE—Marine and Environmental Sciences Centre, Escola Superior de Tecnologia do Barreiro, Instituto Politécnico de Setúbal, Campus do IPS—Estefanilha, 2910-761 Setúbal, Portugal; marta.justino@estbarreiro.ips.pt (M.C.J.); carla.santos@estbarreiro.ips.pt (C.A.S.); 2 Escola Superior de Tecnologia do Barreiro, Instituto Politécnico de Setúbal, Rua Américo da Silva Marinho, 2839-001 Lavradio, Portugal; 2024136843@estudantes.ips.pt; 3 Embrapa—Empresa Brasileira de Pesquisa Agropecuária, Parque Estação Biológica (PqEB), s/nº, Brasília CEP 70770-901, DF, Brazil; afojunior@gmail.com

**Keywords:** bacterial metagenomics, Clostridiaceae, *Prevotella paludivivens*, *Oenococcus*, *Lactobacillus*, winery effluents microbiome

## Abstract

The sustainable reuse of agro-industrial effluents requires a detailed understanding of their microbial composition, especially in the context of integrated vineyard–winery ecosystems. This study investigated the bacterial communities present in winery effluents generated during the early stages of red wine production, using samples collected at a winery in the Setúbal Peninsula, Portugal. Metagenomic analysis targeting the 16S rRNA gene was used to characterise microbial diversity and identify taxa with potential relevance for biotechnology and environmental applications. The effluents exhibited a diverse microbiome, including *Prevotella paludivivens*, species from the *Lactobacillus* genus, and members of the Clostridiaceae family, the latter representing about 5% of the total community. Functional profiling of lactic acid bacteria revealed the predominance of *Oenococcus* and *Lactobacillus* genera, highlighting adaptive traits that may be beneficial under stress conditions. These results suggest that winery effluents, often considered waste, harbour microbial communities with functional potential that extends beyond fermentation, contributing to a broader grape–wine microbial system. The findings emphasise the value of studying winemaking byproducts as reservoirs of microbial diversity and as resources for developing innovative and sustainable applications in biotechnology and environmental management within the wine industry.

## 1. Introduction

Water scarcity is a critical global issue, affecting billions of people worldwide, with four billion experiencing severe water shortages for at least one month each year [1]. This growing challenge underscores the urgent need to build resilient agricultural systems, especially in vulnerable regions where water demand is rapidly increasing. Within the Iberian Peninsula, Portugal exemplifies these pressures, particularly in regions such as Setúbal, where expanding agricultural areas and intensified farming practices have significantly raised water consumption [2]. This situation highlights the close interconnection between water availability and food security, emphasising the importance of implementing sustainable water management strategies at both local and regional levels. Amidst these agro-industrial challenges, Portugal’s wine heritage stands out due to its longstanding tradition in the country. The wine industry’s reliance on water further accentuates the importance of effective water management to ensure the continuity of this vital sector [3,4,5,6]. Efforts to address water scarcity and promote sustainable water use are therefore crucial, not only for safeguarding agricultural productivity but also for preserving cultural traditions such as winemaking.

Against this backdrop, there is growing interest in water reuse as a sustainable practice, especially in agriculture, the largest global water consumer. Advances in wastewater treatment technologies have enabled the production of water at varying quality standards for diverse applications. However, recent global reviews reveal significant gaps and discrepancies in agricultural water reuse regulations, particularly concerning emerging pollutants and treatment harmonisation [7]. While these efforts primarily focus on human health and environmental protection, agro-industrial effluents, such as those from wine production, also represent complex microbial ecosystems. Exploring the microbial communities present in winery effluents is one of the innovative approaches embraced by the REDWine project (www.redwineproject.eu, accessed on 30 July 2025), which aims to develop a circular and sustainable business model by maximising the use of all residues generated during wine production while also creating new opportunities to diversify revenue streams for wine producers. In this context, identifying the microorganisms present in winery effluents is an important step, as it contributes to a better understanding of the impact of effluent disposal or treatment, and may reveal innovative approaches with potential biotechnological value.

The winery wastewater variability stems from fluctuating water volumes, which can be attributed to varying volumes of water used for cleaning the wine production facilities/equipment, unpredictable weather conditions, differences in grape varieties and their unique qualities across different wine-producing regions, as well as variations in local soil and land characteristics [8]. Despite this complex interplay of multiple factors, winery effluents consistently exhibit high concentrations of organic matter and total suspended solids, along with an acidic character [9,10,11,12]. Indeed, the physicochemical characterisation of effluents obtained throughout the wine campaign revealed that the chemical nature of the compounds present in the solution remains constant, varying in their concentration [13]. This physicochemical environment not only creates the conditions for the formation of a specific microbiome but also plays a role in shaping that microbiome diversity, influencing aspects such as species richness and evenness [14,15]. Being a fermented product, wine naturally hosts a diverse array of microorganisms tolerant to both acids and ethanol. In addition to the wine yeast *Saccharomyces cerevisiae* and the bacterium responsible for malolactic fermentation, the *Oenococcus oeni*, various lactic acid bacteria genera such as *Lactobacillus*, *Pediococcus*, and *Leuconostoc* have been reported as inhabitants in both must and wine [16,17,18]. Also, *Clostridium*, being one of the largest bacterial genera, and commonly found in soil, water, and in the gastrointestinal tracts of animals, is frequently reported in agro-industrial effluents. The study of microbiomes, particularly in complex environments like agro-industrial wastewater, requires interdisciplinary approaches in molecular biology and bioinformatics. Methods such as next-generation sequencing (NGS) excel in this field, enabling rapid, accurate, and comprehensive analysis of millions of DNA fragments [19]. In that regard, metagenomic sequencing analysis, a technical application of bioinformatics, provides an unbiased view of microbial diversity and expands the knowledge related to genetic diversity, population structure, and the ecological role of microorganisms [20,21]. These sophisticated techniques complement the NGS methodology by providing insights into the functional potential of microbial communities, thereby enhancing the comprehension of their ecological roles and biotechnological applications. Functional metagenomics, in turn, becomes essential for uncovering the functional genes present in these microbial communities. By leveraging comprehensive databases, this approach can effectively identify pathways involved in the degradation of compounds found in effluents and assess the potential of microorganisms for various applications. The functional insight encompasses details about metabolic processes, transport mechanisms, transcription activities, and carbohydrate metabolism, providing a more comprehensive analysis beyond microbial diversity and composition alone [3,5,20,21,22].

This study aimed to characterise the bacterial community present in effluents from red wine production through 16S rRNA sequencing. By analysing samples collected weekly during the production campaign at a winery in Setúbal, the goal was to better understand the microbial diversity shaped by the variable composition of winery wastewater and its implications for environmental impact and biotechnological potential.

## 2. Materials and Methods

### 2.1. Sample Characterisation

Three individual samples were collected from the effluent tank over three consecutive weeks during one wine campaign. The effluents were obtained at a winery in the Peninsula of Setúbal region and originated from the initial operations of the winemaking process, comprising grape reception, destemming, and equipment cleaning, thus representing the natural variability over the fermentation season. Each effluent sample was identified by the code “RWDDMMYY”, where “RW” denotes red wine, and “DD”, “MM”, and “YY” represent the day, month, and year of collection, respectively. Approximately 1 L of each sample was collected in clean containers and stored at −20 °C until further analysis. DNA extraction was performed using the NZYSoil gDNA isolation kit (NZYTech MB21802), following the manufacturer’s instructions for water samples. The processed effluent volume ranged from 40 to 100 mL, yielding a pellet mass between 250 and 500 mg. This adjustment was necessary due to the variable suspended-solids load of winery effluents, to obtain pellet masses within the manufacturer’s recommended range and to avoid filter/column overloading. Centrifugation parameters were kept constant for all samples at 4 °C, 4900 rpm for 10 min. DNA was quantified spectrophotometrically, and a fixed amount of DNA was used as a template for PCR. DNA was quantified in a μDrop plate with a Multiskan SkyHigh Microplate Spectrophotometer (from ThermoFisher, Waltham, MA, USA). DNA quality was evaluated based on A260/280 (1.8–2.0) and A260/230 (expected >1.8). Physicochemical analysis of effluent samples was performed for pH, conductivity, total sugars [23], and reducing sugars [24]. Also, the total phenolic content was determined using the Folin–Ciocalteu reagent method, with gallic acid as the standard [25]. These measurements were performed in technical triplicates (*n* = 3) for each biological sample.

### 2.2. Sequencing and Metagenomics

The V3–V4 region of the 16S rRNA gene was amplified using primers 341F (5′-CCTACGGGNGGCWGCAG-3′) and 785R (5′-GACTACHVGGGTATCTAATCC-3′), as described by Fadeev et al. (2021) [26]. Library preparation followed the Illumina 16S Metagenomic Sequencing Library Prep protocol (Illumina, 15044223 Rev. B). Sequencing was performed by Stabvida, Lda (Almada, Portugal) on an Illumina MiSeq platform using the MiSeq Reagent Kit v3, generating 2 × 300 bp paired-end reads. Quality control of the raw sequence data was assessed with FastQC, confirming that more than 60% of bases had Phred quality scores above Q30.

DNA concentration was measured with a Qubit 2.0 fluorometer (Thermo Fisher Scientific, Waltham, MA, USA) using the Qubit dsDNA BR Assay Kit, and DNA integrity was evaluated by 1.5% agarose gel electrophoresis in TAE buffer. Bioinformatic processing was conducted in QIIME2 (version 2022.2) [27]. Reads were denoised with the DADA2 plugin, which included trimming, dereplication and chimaera filtering. Sequences were then clustered into operational taxonomic units (OTUs) at 99% similarity. Taxonomic assignment was performed with a scikit-learn classifier trained on the SILVA 138 reference database, trimmed to the V3–V4 region, using a confidence threshold of 0.7. Only OTUs represented by ≥10 reads were retained for downstream analyses.

To ensure comparability between samples, taxonomic assignments were performed against a consistent reference database, applying uniform thresholds for quality filtering and classification confidence. Relative abundances were then calculated at each taxonomic level, and the resulting data were visualised using identical settings in the Sankey diagram tool. This standardised approach ensured that the differences observed across samples reflected true variations in microbial community composition, rather than methodological inconsistencies. Microbial species’ richness, abundance, and evenness were evaluated by the alpha rarefaction curve.

### 2.3. Functional Comparative Genomic Analysis

Representative genomes of lactic acid bacteria commonly associated with wine fermentation (*Oenococcus* spp. and related *Lactobacillus* lineages) were selected for functional comparison. These included publicly available reference genomes of lactic acid bacteria retrieved from NCBI RefSeq/GenBank. The dataset included *Secundilactobacillus paracollinoides* TMW 1.1995 (syn. *Lactobacillus paracollinoides*; GCA_001702195.1; CP014924–CP014932), *Liquorilactobacillus mali* LM596 (syn. *Lactobacillus mali*; GCF_009184705.1), *Schleiferilactobacillus harbinensis* M1 (syn. *Lactobacillus harbinensis*; GCF_009217765.1), *Lentilactobacillus parabuchneri* FAM21731 (syn. *Lactobacillus parabuchneri*; GCF_001922025.1; CP018796–CP018797), *Liquorilactobacillus nagelii* TMW 1.1827 (syn. *Lactobacillus nagelii*; GCA_002850055.1; CP018180–CP018183), *Oenococcus oeni* AWRIB787 (GCA_020424345.1) and *Oenococcus* sp. UCMA 16435 (GCF_004010835.2). All genomes were accessed in August 2025. Genomes were selected to represent strains from related fermentation environments and to enable comparisons within *Oenococcus* and *Lactobacillus* lineages. Protein families were annotated using the PGFam classification system within the Bacterial and Viral Bioinformatics Resource Centre (BV-BRC, version 3.53.3) [28], applying default parameters. Pairwise BLAST analyses, using the standalone BLAST+ suite, version 2.13.0 (NCBI, Bethesda, MD, USA) were conducted with a threshold of ≥95% sequence identity and ≥90% alignment coverage, considering only hits with e-values < 1 × 10^−5^. Additional functional exploration was performed using the Protein Family Sorter and Comparative Pathways Viewer within BV-BRC to identify pathways, subsystems, and protein families.

## 3. Results and Discussion

### 3.1. Physicochemical Characterisation of Red Wine Effluents

Previous studies have shown that the physicochemical composition of winery effluents is highly variable, particularly in terms of organic matter, such as sugars and polyphenols, which can fluctuate significantly over three consecutive weeks of the wine campaign [9,11,29,30,31]. Reported sugar concentrations ranged from 0.13 to 107.89 g·L^−1^, while total phenolic compounds load varied between 0.001 and 0.531 g·L^−1^ [31]. This variability can play a key role in shaping the specific microbiome that develops in these effluents, reinforcing the importance of their characterisation when exploring potential biotechnological applications.

In line with these findings, the effluent samples analysed in this study exhibited typical features observed in comparable wastewater streams [13]. These characteristics often include acidic pH levels and a substantial presence of organic carbon compounds, particularly sugars like glucose and fructose, commonly found in grapes. Additionally, certain parameters displayed evident fluctuations, reflecting the dynamic nature of winemaking processes. Specifically, red wine effluents showed higher phenolic content in the late September sample and large differences in sugar concentrations across weeks, as outlined in Table 1. In contrast, conductivity values remained relatively stable across samples.

The first week’s sample of the 2021 campaign (RW080921) had the highest sugar content, serving as a rich carbon and energy source for many microorganisms. The remaining samples exhibited approximately 30 times fewer reducing sugars and 16 times fewer total sugars.

Phenolic content regarding polyphenols in the effluents may exert dual effects on microbial populations. On one hand, they act as antioxidants, neutralising reactive oxygen species and protecting against oxidative damage [32]. On the other hand, they may function as anti-nutrients, interfering with microbial metabolism, reducing nutrient bioavailability, and hindering microbial activity [33]. Nevertheless, our analysis was only based on total phenolic content, so the contribution of specific compounds cannot be assessed here.

### 3.2. Bacterial Taxonomic Identification

#### 3.2.1. Overview of the Bacterial Community in the Composite Effluent Sample

**Figure 1:**
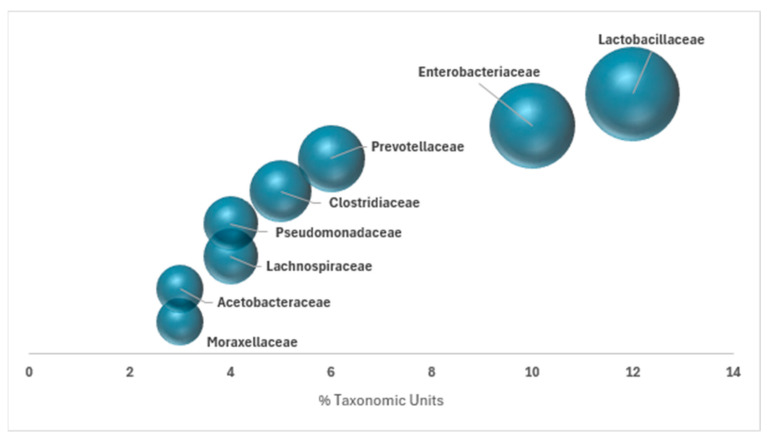
Relative abundance of bacterial taxa in winery effluent samples (RW080921, RW160921, RW270921). Only taxa with relative abundance greater than 2% are shown. Taxonomic assignment was performed using QIIME2 (v2022.2) with a scikit-learn classifier trained on the SILVA 138 database at 99% similarity (confidence threshold 0.7).

Overall, a comprehensive metagenomic analysis was performed on a pooled sample comprising all effluents collected during the September wine campaign. This integrated approach aimed to provide a broad overview of the microbial diversity present across the three sampling weeks over the same campaign. From these three samples, a total of 450 operational taxonomic units (OTUs) were identified, representing groups of phylogenetically related microorganisms. The results revealed a marked diversity in the bacterial community, with notable predominance of the families Lactobacillaceae and Enterobacteriaceae, accounting for 12% and 10% of the relative OTU abundance, respectively. The most prevalent bacterial families, those exceeding 2% in relative abundance, are illustrated in Figure 1 and together represent 47% of the total detected microbial population.

**Figure 2:**
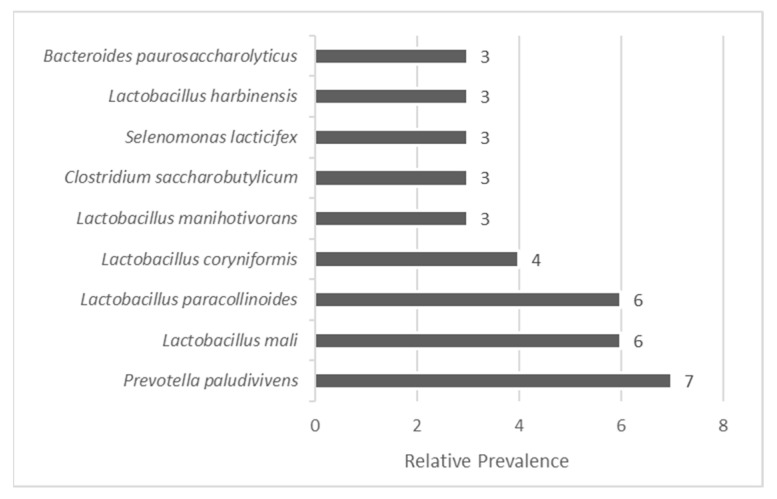
Most prevalent bacterial taxa (relative abundance greater than 2%) in the winery effluent samples. Taxa were identified after denoising and chimaera removal with DADA2 in QIIME2, and clustered into OTUs at 99% similarity using the SILVA 138 reference database. Species-level assignments shown correspond to database predictions and should be interpreted with caution.

The analysis of the most prevalent bacterial species, defined as those with a relative abundance greater than 2%, revealed several microorganisms frequently associated with fermentation environments. While their ecological role in winery effluents is evident, potential applications would require further functional validation. This threshold, which considers only species that represent more than 2% of the total sequences assigned to operational taxonomic units (OTUs), was selected to focus the analysis on dominant taxa that are more likely to exert functional influence within the microbial ecosystem (Figure 2). By filtering out low-abundance species, this approach allows a clearer interpretation of the community’s active contributors and their possible applications.

Addressing the specific species identified (87 in total; Figure 2), *Prevotella paludivivens* and five *Lactobacillus* species emerged as the most prevalent bacterial taxa in the pooled effluent sample. Notably, *Prevotella paludivivens* stands out as one of the few species within the *Prevotella* genus that originates from natural habitats rather than mammalian microbiota. It is capable of degrading various polysaccharides, producing metabolic end-products such as acetate and succinate, which are of interest for biofuel production and bioprocessing applications [34,35].

Within the *Lactobacillus* genus, *Lactobacillus mali* and *Lactobacillus paracollinoides* exhibited the highest incidence. These species are well documented in the literature for their contributions to food preservation and flavour development. Moreover, they have potential health benefits through their probiotic activity and antimicrobial properties [36]. *Lactobacillus mali*, in particular, has demonstrated potential in the management of metabolic syndrome, including obesity and diabetes-related conditions [37,38]. *Lactobacillus coryniformis*, also present in the sample, is known to produce exopolysaccharides with antioxidant and antibacterial activities, with possible applications in gastrointestinal health products, vaccine adjuvants, and as additives in animal feed [39].

In addition to fermentative bacteria, *Clostridium saccharobutylicum* was detected. Like other *Clostridium* species, it plays a key role in the degradation of complex biomass such as lignocellulose and has been explored for its potential in the renewable production of biofuels and biosolvents, particularly when used in co-culture strategies [40].

Finally, *Bacteroides paurosaccharolyticus*, a strictly anaerobic bacterium, was also identified. This species can metabolise a broad spectrum of sugars into short-chain fatty acids (SCFAs) such as propionate, acetate, and succinate [41], making it a valuable candidate for biofuel production and other industrial bioconversion processes [42].

Together, these findings highlight a metabolically versatile and functionally relevant microbial consortium within winery effluents, suggesting potential for valorisation strategies in environmental biotechnology, waste treatment, and bioproduct generation.

#### 3.2.2. Taxonomic Profiles of Individual Effluent Samples

The taxonomic profiles of the individual effluent samples revealed distinct microbial communities shaped by the specific composition of each effluent. Among the key factors with potential to influence these microbial dynamics are the concentrations of sugars and polyphenols, two components that varied considerably across samples. Simple and fermentable sugars provide readily available carbon sources, which may favour the growth of fermentative and fast-growing bacterial genera such as *Lactobacillus* and members of the Enterobacteriaceae family. In contrast, polyphenols, also abundant in winemaking residues, are known for their antimicrobial properties, capable of exerting selective pressure on microbial populations. While inhibitory to some taxa, these compounds may enable the persistence or proliferation of bacteria with specific tolerance mechanisms or metabolic adaptations.

**Figure 3:**
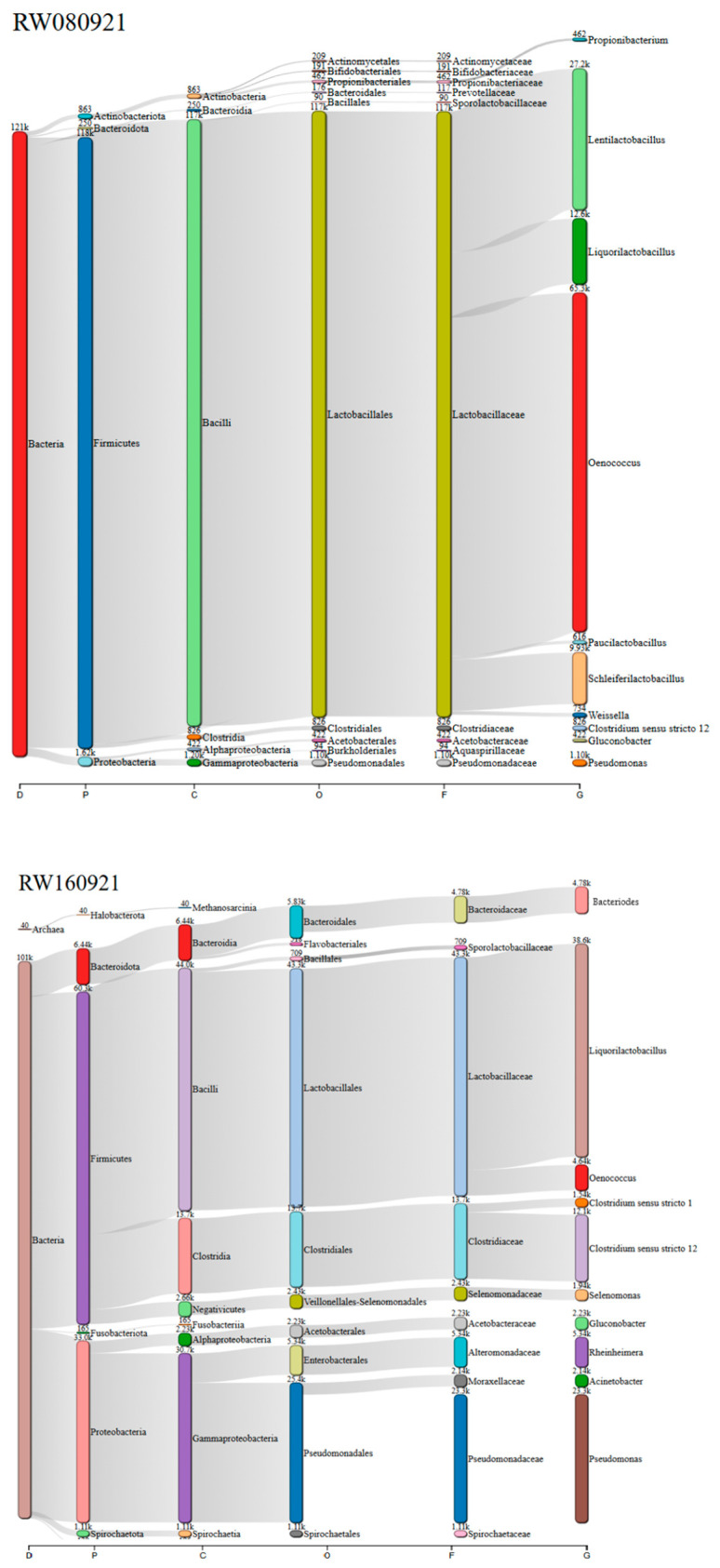

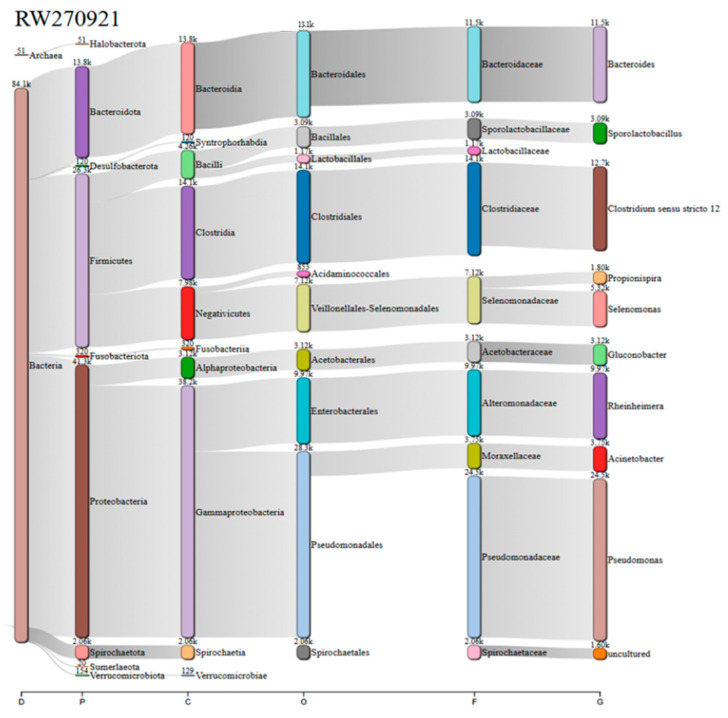
Sankey diagrams showing the taxonomic distribution of bacterial communities across individual effluent samples from different weeks in the same wine campaign (RW080921, RW160921, RW270921). Taxonomic ranks are indicated as follows: D (domain), P (phylum), C (class), O (order), F (family), G (genus). Relative abundances were calculated based on filtered OTUs (≥10 reads).

To visualise the taxonomic distribution of sequences within each effluent sample, Sankey (alluvial) diagrams were employed. These diagrams illustrate the distribution of classified reads across taxonomic levels, providing a clear and sample-specific representation of the microbial structure. In the context of this study, this approach enabled the identification of dominant microbial groups and supported the comparison of taxonomic profiles in relation to the compositional variability of the effluents. The resulting visualisations are presented in Figure 3.

The Sankey diagrams effectively illustrate how the composition and richness of bacterial taxa varied from sample to sample, highlighting shifts in the relative abundance of dominant groups. It becomes apparent that each effluent harboured a distinct microbial profile, reflecting the heterogeneous nature of winery effluents. In the RW080921 sample, the bacterial community was mainly composed of *Oenococcus* and *Lactobacillus* genera, characteristic lactic acid bacteria associated with early stages of grape processing. In contrast, the RW160921 sample showed a reduction in *Oenococcus* and a notable increase in microbial diversity, with the emergence of genera such as *Clostridium* and *Pediococcus*, as well as bacteria belonging to the family Enterobacteriaceae. Additionally, species of the genus *Pseudomonas* were detected in this sample for the first time. In the subsequent RW270921 sample, the microbial profile shifted further, becoming dominated by Enterobacteriaceae and *Clostridium*, while lactic acid bacteria were no longer prevalent. The increased presence of *Pseudomonas* species in this final sample is particularly noteworthy, as members of this genus are known for their metabolic versatility and capacity to degrade a wide range of organic compounds, including environmental pollutants. Their enrichment may reflect adaptive responses to the more complex or recalcitrant chemical composition of later-stage winery effluents, potentially influencing downstream ecological interactions and bioremediation processes. The increasing prevalence of *Enterobacteriaceae* across the RW160921 and RW270921 samples is also of environmental relevance. This family includes a wide range of facultative anaerobic Gram-negative bacteria, some of which are commonly associated with faecal contamination or opportunistic pathogenicity. Their presence in winery effluents may indicate external contamination or proliferation under anaerobic, nutrient-rich conditions. In environmental settings, these bacteria can contribute to the degradation of organic matter, but they also pose potential risks if released untreated into natural ecosystems, where they may disrupt native microbial communities, impact water quality, or carry antibiotic resistance genes [43].

**Figure 4:**
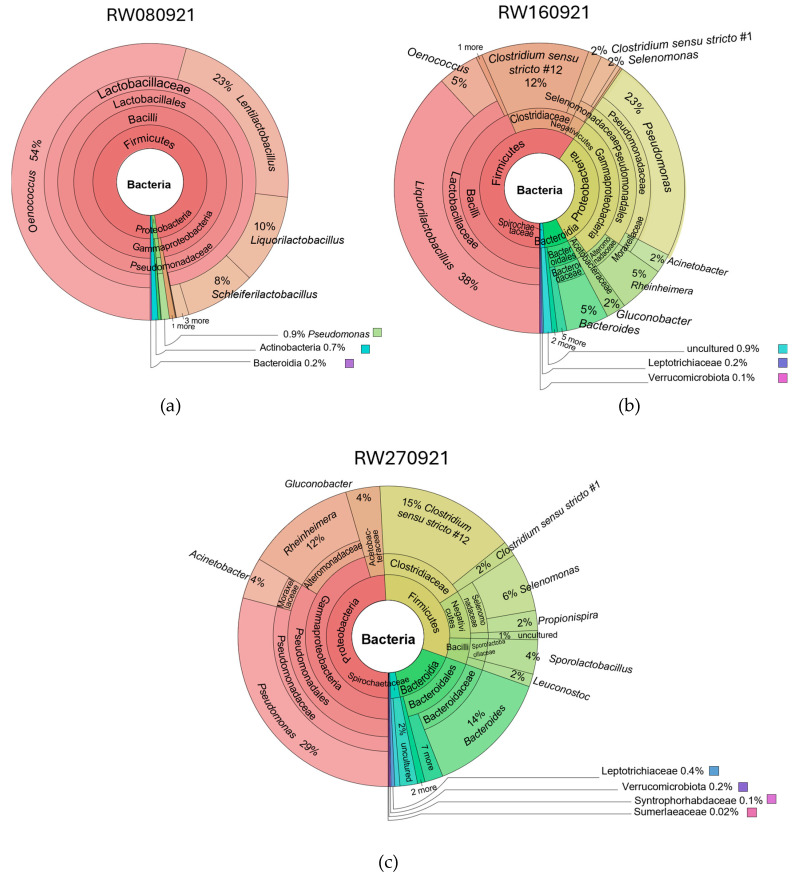
Krona charts illustrating bacterial richness and relative abundance in winery effluent samples. (**a**) RW080921, (**b**) RW160921, and (**c**) RW270921. Charts display the hierarchical distribution of taxa from domain to species level, with relative proportions represented by sector size.

As an additional tool to enhance the quantification and visualisation of bacterial populations in each effluent sample, Krona charts were generated (Figure 4). This format enables a more detailed inspection of the microbial community by illustrating the full spectrum of taxa present in each sample, from domain to species level. The interactive nature of Krona charts, captured here as static snapshots, also facilitates intuitive comparison between samples, particularly in terms of richness and dominance of specific bacterial groups.

The Krona chart analysis of the RW080921 (Figure 4a) sample reveals a clear dominance of Gram-positive lactic acid bacteria (LAB), with *Oenococcus* representing 54% of the total bacterial community and *Lactobacillus* accounting for 41%. Within the latter group, 23% correspond to *Lentilactobacillus*, 10% to *Liquorilactobacillus*, and 8% to *Schleiferilactobacillus*. These LAB are naturally associated with grape surfaces and soil, and their high relative abundance is consistent with the nature of the effluent, which was collected during the grape reception and destemming stages—key points where indigenous microbiota from the fruit enter the winery system.

*Oenococcus*, in particular, plays a crucial role in winemaking by catalysing malolactic fermentation (MLF), a process that converts L-malic acid into L-lactic acid and carbon dioxide, contributing to wine stability and sensory refinement [44]. The notable presence of LAB such as *Lentilactobacillus*, *Liquorilactobacillus*, *Schleiferilactobacillus*, and *Oenococcus* in winery effluents is not only expected but also environmentally unproblematic. These genera, previously grouped under the broader *Lactobacillus* classification, are well known for their roles in food fermentation and are considered beneficial microorganisms [45].

Importantly, these LAB are regarded as non-pathogenic and safe for both human health and the environment. As highlighted by Bernardeau et al. (2008) [46], ingestion of *Lactobacillus* species is generally not hazardous, with rare cases of infection occurring only in predisposed individuals. This reinforces the view that their presence in winery effluents poses no significant public health risk and may even support microbial balance and natural biodegradation processes in effluent-impacted environments.

Additionally, the Krona chart of the RW080921 enabled the identification of low-abundance genera such as *Pseudomonas* (0.9%), whose presence was only subtly suggested in the Sankey diagram due to its qualitative rather than quantitative nature.

The Krona chart analysis of the RW160921 (Figure 4b) sample revealed a bacterial community composed primarily of *Liquorilactobacillus* (38%), *Pseudomonas* (23%), *Clostridium* (14%), *Oenococcus* (5%), and *Acinetobacter* (2%).

As previously observed in the RW080921 sample, *Liquorilactobacillus* and *Oenococcus* are lactic acid bacteria (LAB) commonly found in fermentation environments such as winemaking. They contribute to natural biodegradation and malolactic fermentation, which helps in the transformation of malic acid into lactic acid, gently modifying wine acidity. These bacteria are generally regarded as safe and are typical inhabitants of winery environments [46].

*Clostridium* spp. constitute a large and heterogeneous genus of obligate anaerobes classified into several clusters. Members of the genus *Clostridium* were detected. This genus is taxonomically diverse and includes both commensal and pathogenic species. Given the limited resolution of 16S rRNA (V3–V4) sequencing, our results can only confirm presence at the genus level, and no inference regarding pathogenic potential at the species level can be made. Their presence in winery effluents likely reflects their metabolic versatility and role in anaerobic organic matter degradation, contributing positively to biodegradation processes [47].

*Pseudomonas* species are common soil and water bacteria known for their metabolic diversity and ability to degrade a wide range of organic compounds. Their relatively high abundance in this sample suggests an active role in biodegradation within the winery effluent environment.

Finally, *Acinetobacter* spp., Gram-negative coccobacilli belonging to the family Moraxellaceae within the order Pseudomonadales, were detected at low relative abundance (2%). Although some species, notably Acinetobacter baumannii, are recognised as opportunistic pathogens with multidrug-resistant strains that pose significant public health challenges, their low prevalence here implies a minimal risk in this context [48].

Ultimately, The Krona chart analysis of the RW270921 (Figure 4c) sample revealed a bacterial community composed mainly of *Pseudomonas* (29%), *Clostridium* (17%), *Rheinheimera* (12%), *Sporolactobacillus* (14%), *Bacteroides* (6%), *Acinetobacter* (4%), *Gluconobacter* (4%), and *Selenomonas* (4%). Compared to the previous sample, there is an increase in the relative abundance of *Pseudomonas*, *Acinetobacter* spp., and *Clostridium*, which may indicate shifts in environmental conditions favouring these genera or changes in biodegradation dynamics within the effluent. As noted previously, the possibility of opportunistic pathogenic species among these bacteria necessitates a full assessment of potential risks [47,48].

The genus *Rheinheimera* comprises Gram-negative, flagellated, rod-shaped to coccoid bacteria that are oxidase- and catalase-positive, aerobic, and chemoheterotrophic. Currently, it includes eight recognised species isolated from diverse environments such as seawater, freshwater, soil, and plant roots, indicating a wide ecological distribution [49]. These bacteria are primarily known for their role in organic matter degradation and, in some cases, for producing antimicrobial compounds, suggesting a beneficial functional profile in the environment.

The presence of *Bacteroides*, typically associated with organic matter decomposition, further underscores the complex and dynamic microbial ecology of the winery effluent. Other genera, such as *Gluconobacter*, *Selenomonas*, and *Sporolactobacillus,* are generally linked to fermentation and nutrient cycling, playing supportive and environmentally benign roles within the microbial community of winery effluents. Their presence does not reflect significant environmental risks.

By correlating the diversity of these species with the physicochemical composition of the effluents (Table 1), it is plausible to hypothesise that the dominance of LAB species in sample RW080921 could be associated with a higher concentration of total sugars, which likely favours species adapted to malolactic fermentation. As sugar levels decrease, these particular species may lose their competitive edge relative to others that were initially present but less dominant. Conversely, the elevated concentration of polyphenols seems to potentially facilitate the growth of Enterobacteriaceae and Clostridiaceae bacteria. Also, from Figure 4, it was possible to corroborate the results from Pinto and coworkers who found that bacterial communities involved in wine production presented a multifaceted composition, with Firmicutes and Proteobacteria as predominant phyla within the prokaryotic population [50].

#### 3.2.3. Genome-Based Functional Analysis of Bacterial Communities in Winery Effluents

According to the results depicted in Figure 4, the RW080921 sample was identified as resembling a vineyard sample based on the predominant bacterial families detected. Consequently, this sample was selected to advance to the bioinformatic analysis. Aligning sequences from the genera *Oenococcus* and *Lactobacillus* against the NCBI database using the BLASTN tool [51] revealed that the DNA primarily belonged to species *O. sicerae* (95%) and *O. oeni* (0.5%). These species are commonly associated with wine and cider production [52,53], reinforcing their relevance within the studied sample.

The bacterial genome-based proteome analysis, utilising bidirectional BLASTP for protein sequence-based genome comparison, was conducted using the obtained genomic data and submitted to the Bacterial and Viral Bioinformatics Resource Centre (BV-BRC) database, also known as PATRIC. Genomes of the identified species were analysed to predict the patterns of functional sequences (proteins). Up to seven genomes of species present in the first sample were compared to a reference genome, specifically *L. paracollinoides*, selected due to its high prevalence in both the initial sample and the overall analysis.

**Figure 5:**
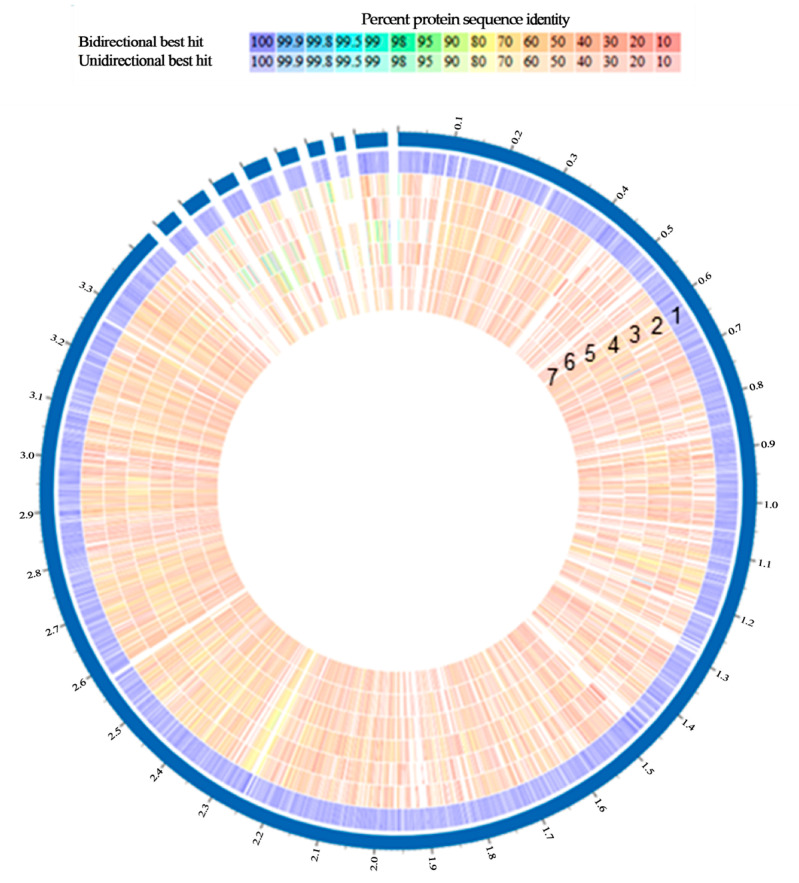
Circular genome comparison of lactic acid bacteria isolates using BV-BRC comparative genomics tools. Tracks (from outer to inner) correspond to (1) *Secundilactobacillus paracollinoides* TMW 1.1995 (GCA_001702195.1), (2) *Liquorilactobacillus mali* LM596 (GCF_009184705.1), (3) *Schleiferilactobacillus harbinensis* M1 (GCF_009217765.1), (4) *Lentilactobacillus parabuchneri* FAM21731 (GCF_001922025.1), (5) *Liquorilactobacillus nagelii* TMW 1.1827 (GCA_002850055.1), (6) *Oenococcus oeni* AWRIB787 (GCA_020424345.1), (7) *Oenococcus* sp. UCMA 16435 (GCF_004010835.2). Shared protein families (PGFams) are shown as conserved regions across genomes.

The analysis was displayed as a circular genome view (Figure 5), with the outermost line representing the genome of *L. paracollinoides*, used as reference, followed by other *Lactobacillus* species and *Oenococcus* spp. Among the *Lactobacillus* spp. identified in this sample, multiple protein patterns were observed. *L. paracollinoides* stood out due to its distinctly unique profile, exhibiting average similarities below 60%. In contrast, the other *Lactobacillus* spp. showed a higher degree of similarity to each other and to *Oenococcus* spp.

**Figure 6:**
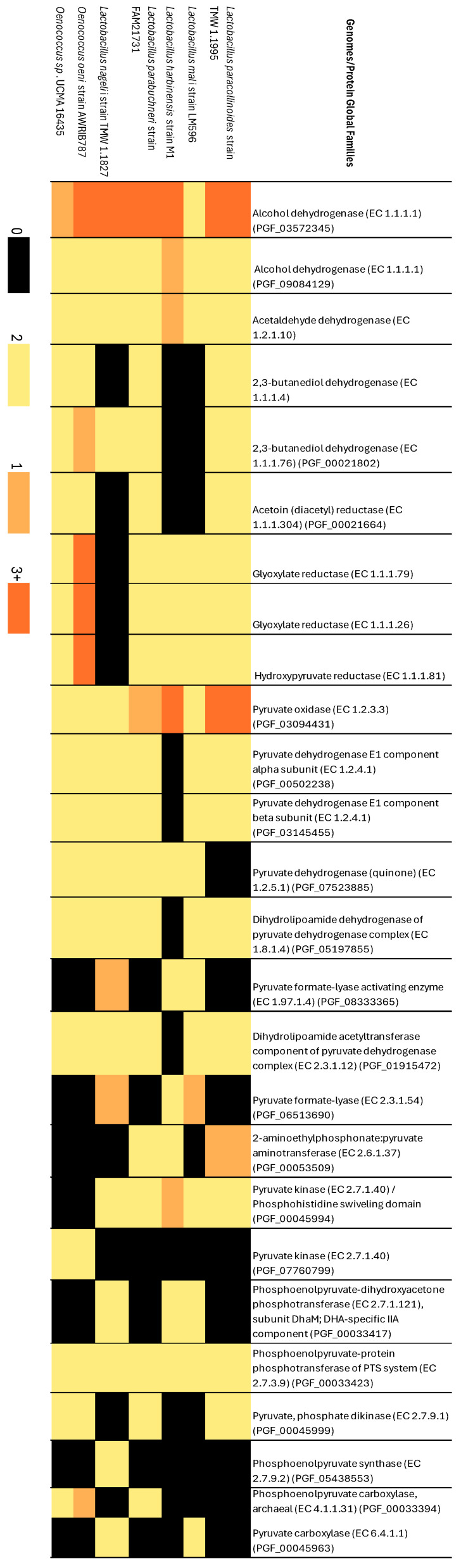
Heatmap of protein global families (PGFams) related to fermentation and glycolysis pathways in lactic acid bacteria genomes. Presence/absence was determined using BV-BRC (v3.53.3) PGFam annotation. Colour intensity represents the number of proteins assigned per genome: black = none, bright yellow = one, dark yellow = two, orange = ≥3. Strains included are the same as in Figure 5.

The PATRIC database was used to compare global families of proteins (PGF) involved in biochemical pathways across a set of LAB bacteria, leveraging their genomic data. This analysis specifically examined pathways associated with alcoholic fermentation, malolactic fermentation, and glycolysis. Alcohol dehydrogenase (EC 1.1.1.1) is ubiquitous across all represented microorganisms, with a higher prevalence observed in *Lactobacillus* spp. It was noted to be categorised into various global protein families among different genes (Figure 6). This genomic diversity results in distinct colour patterns in the heatmap. The protein family acetoin (diacetyl) reductase (EC 1.1.1.304) catalyses the conversion of (S)-acetoin and NAD(+) to diacetyl, H(+), and NADH. This enzyme is consistently linked with the activity of 2,3-butanediol dehydrogenase (EC 1.1.1.4 and EC 1.1.76), which reversibly converts (S)-acetoin to (S, S)-butane-2,3-diol, as shown in previous studies [54,55].

The association between diacetyl reductase and butanediol dehydrogenases plays a crucial role in metabolic pathways, particularly in microbial fermentation processes, which are vital in industries like winemaking. Diacetyl and its reduced product, 2,3-butanediol, significantly influence the flavour and aroma of fermented products, with diacetyl imparting a buttery taste that can harm quality if overproduced. Diacetyl reductase irreversibly reduces diacetyl to 2,3-butanediol, helping manage these levels. Understanding these enzymes can optimise fermentation efficiency, improve yields, and control by-products, while also having biotechnological potential for chemical and pharmaceutical [56].

Moreover, 2,3-butanediol dehydrogenases (EC 1.1.1.4, EC 1.1.1.304, and EC 1.1.1.76) were found to be absent in *Lactobacillus mali* and *Lactobacillus harbinensis*, while EC 1.1.1.76 is present in *Lactobacillus nagelii*. These results suggest that each species of the *Lactobacillus* genus may employ distinct metabolic strategies for carbohydrate fermentation. While certain *Lactobacillus* spp. rely on specific metabolic pathways and protein families for fermentation, others may have developed alternative routes or mechanisms to achieve similar outcomes. Therefore, the absence of certain protein families in some *Lactobacillus* strains compared to *Oenococcus* may indicate divergent evolutionary adaptations among these microorganisms [57].

In glycolysis, the heatmap illustrates annotations for the same protein across various global families in the genome, distinguished by different colours, reflecting its differential presence among microorganisms. For instance, pyruvate kinases (EC 2.7.1.40) from multiple global families show variable presence depending on the genus, emphasising the genetic biodiversity. This variability is evident in the heatmap, where both *Oenococcus* spp. consistently exhibit similar colours across all biochemical pathways, contrasting markedly with *Lactobacillus* bacteria.

Furthermore, the analysis extends to the biochemical pathway responsible for metabolising malic acid. A multiple genome sequence alignment focused on the region annotated as the global protein family of malolactic regulator reveals notable differences between Oenococcus and Lactobacillus. Specifically, *L. paracollinoides*, *L. parabuchneri*, and *L. mali* within the *Lactobacillus* genus do not possess proteins from this annotated global family, in contrast to Oenococcus. These insights emphasise the diverse metabolic strategies within Lactobacillus and Oenococcus bacteria, particularly concerning glycolysis and malic acid metabolism.

## 4. Conclusions

Bacterial metagenomics (16S rRNA) facilitated the taxonomic evaluation of microbial communities, offering insights into the diversity of microbiomes in effluents from red wine-making processes. Moreover, it sheds light on the variability of factors such as species richness and evenness, influenced by the diverse chemical composition of effluents, which includes concentrations of sugars and polyphenols.

The analysis of the bacterial families revealed a multitude of species with significant potential for biotechnological and bioremediation applications. *Prevotella paludivivens* emerged as a standout bacterium within the *Prevotella* genus, displaying promise in polysaccharide decomposition, particularly in lignocellulosic residues, which are a widespread and renewable natural resource, thereby offering the potential for environmentally friendly biofuel production. Species within the *Lactobacillus genus*, such as *Lactobacillus mali*, *Lactobacillus paracollicoides*, and *Lactobacillus coryniformis*, showcased versatile roles. The species *Clostridium saccharobutylicum* exhibits broad and flexible metabolic capabilities, serving as a solventogenic bacterium applied in the industrial production of acetone, butanol, and ethanol.

Within the realm of lactic acid bacteria (LAB), renowned for their production of lactic acid as a primary metabolic byproduct of carbohydrate fermentation, the most prevalent genera identified in the effluents were *Oenococcus* and *Lactobacillus*. Subsequent analysis of the data obtained via metagenomic methodologies involved comparative functional genomics techniques, which unveiled that the protein sequences of these species demonstrate approximately 60% identity and similarity in their alignments. The genome analysis confirmed the presence of enzymes associated with alcoholic and malolactic fermentation, although with expected structural differences. The exploration of biochemical pathways and protein families suggested possible structural interrelationships among protein sequences, enriching the understanding of microbial metabolism and adaptation.

In the literature, metagenomic analysis of effluents is typically associated with bioremediation processes, rather than the valorisation of effluents as a source of microorganisms. This study, by highlighting the presence of various microorganisms with diverse biotechnological potential, unveils a new perspective on these effluents. It suggests that effluents can serve not only as targets for treatment but also as valuable reservoirs of microorganisms with applications in biotechnology.

## Figures and Tables

**Table 1:** Physicochemical parameters of winery effluent samples collected on three dates during September 2021 (RW080921, RW160921, RW270921). Values are shown as mean ± standard deviation (*n* = 3 technical replicates). pH and conductivity were measured using a calibrated multiparameter, sugars were quantified by the phenol–sulphuric acid method, and total phenolics by the Folin–Ciocalteu assay (expressed as gallic acid equivalents, GAE).

Sample ID	pH	Conductivity (mS/cm)	Reducing Sugars (g/L)	Total Sugars (g/L)	Total Phenolic Content (g/L)
RW080921	4.57	1.46	4.45 ± 0.25	8.61 ± 0.02	0.195 ± 0.002
RW160921	4.68	0.99	0.13 ± 0.00	0.55 ± 0.00	0.122 ± 0.003
RW270921	4.36	1.59	0.17 ± 0.00	0.53 ± 0.00	0.251 ± 0.003

## Data Availability

The original contributions presented in this study are included in the article. Further inquiries can be directed to the corresponding authors.

## Abbreviations

The following abbreviations are used in this manuscript:

BLAST	Basic Local Alignment Search Tool
BV-BRC	Bacterial and Viral Bioinformatics Resource Centre
LAB	Lactic acid bacteria
MLF	Malolactic fermentation
NGS	Next-generation sequencing
OTU	Operational Taxonomic Units
PGF	Global families of proteins
RW	Red wine
SCFA	Short-chain fatty acid
